# Corona With Lyme: A Long COVID Case Study

**DOI:** 10.7759/cureus.36624

**Published:** 2023-03-24

**Authors:** Danielle C Thor, Sergio Suarez

**Affiliations:** 1 Internal Medicine, Touro College of Osteopathic Medicine, New York, USA; 2 Osteopathic Medicine, Touro College of Osteopathic Medicine, New York, USA

**Keywords:** dysautonomia, covid long haul syndrome, pots, me/cfs, post-viral illness, lyme disease, pacs, post-covid syndrome, long covid, covid-19

## Abstract

The longevity of the coronavirus disease 2019 (COVID-19) pandemic has necessitated continued discussion about the long-term impacts of SARS-CoV-2 infection. Many who develop an acute COVID-19 infection will later face a constellation of enduring symptoms of varying severity, otherwise known as long COVID. As the pandemic reaches its inevitable endemicity, the long COVID patient population will undoubtedly grow and require improved recognition and management. The case presented describes the three-year arc of a previously healthy 26-year-old female medical student from initial infection and induction of long COVID symptomology to near-total remission of the disease. In doing so, the course of this unique post-viral illness and the trials and errors of myriad treatment options will be chronologized, thereby contributing to the continued demand for understanding this mystifying disease.

## Introduction

As the coronavirus disease 2019 (COVID-19) global pandemic enters its third year, its persistence will undoubtedly result in a sustained rise in the population of patients suffering from its unique post-viral illness syndrome, long COVID. Current estimates of the prevalence of long COVID suggest nearly half of all hospitalized patients and a third of all non-hospitalized patients who are infected with SARS-CoV-2 will endure long-term sequelae regardless of a symptomatic or asymptomatic initial infection [1]. The World Health Organization provides a consistent definition for these long-term sequelae included under the long COVID umbrella:

"Post COVID-19 condition occurs in individuals with a history of probable or confirmed SARS CoV-2 infection, usually 3 months from the onset of COVID-19 with symptoms and that last for at least 2 months and cannot be explained by an alternative diagnosis. Common symptoms include fatigue, shortness of breath, cognitive dysfunction but also others and generally have an impact on everyday functioning. Symptoms may be new onset following initial recovery from an acute COVID-19 episode or persist from the initial illness. Symptoms may also fluctuate or relapse over time” [2].

The pathophysiology behind long COVID remains primarily theoretical in its understanding, although recent data have identified promising correlations for further investigation, including specific immunoglobulin signatures, viral titers, and/or autoantibodies associated with the development of the syndrome [3]. The overarching presumption of long COVID resulting from a hyperinflammatory state induced by SARS-CoV-2 infection has spurred a series of discussions on how this mechanism results in symptomatic injury. Such theories include direct neurovascular damage from the virus itself, embolic damage from the hypercoagulable states commonly induced by acute COVID-19 infection, microbiota dysfunction, or secondary autoimmune destruction brought about by this excessive inflammatory response. Comparisons are also commonly drawn between long COVID and several poorly understood chronic conditions with coinciding symptoms, such as myalgic encephalomyelitis and/or chronic fatigue syndrome (ME/CFS), postural orthostatic hypotension syndrome (POTS) and/or dysautonomia, and mast cell activation syndrome (MCAS) [4].

Despite this increase in awareness and well-intended discussion, millions of patients are currently or may soon suffer from long COVID and require more immediate attention from physicians throughout the spectrum of medical practice. Superficially, the deteriorations in the health of these patients have already resulted in significant decreases in productivity and/or quality of life while simultaneously exacerbating the inequities that plague our healthcare systems. More specifically, the “long COVID diagnosis” is laden with deeper controversy, as many “chronic-fatigue-like” syndromes are often met with skepticism by misinformed healthcare providers. Therefore, it is imperative to understand and legitimize both the pathophysiology and patient experience of long COVID while advocating for continued investment in research of viable treatment options.

The following case report details an otherwise healthy and health-literate medical student’s journey with long COVID from the pandemic’s earliest stages to the present day. It intends to contribute to the demand for knowledge and understanding of this unique post-viral illness as a genuine and far-reaching medical syndrome. It will also seek to denote individual successes and failures in recognizing and treating long COVID, including misdiagnoses surrounding a concurrent acute Lyme disease infection, as well as outline future directions in its management.

## Case presentation

A previously healthy 26-year-old female medical student living in New York, NY, was one of the first of her colleagues to be symptomatically infected with the then-novel coronavirus, SARS-CoV-2, in March 2020. Although COVID-19 testing was not available at the time to non-hospitalized patients, the presumed diagnosis of acute COVID-19 infection was confirmed in April 2020 during volunteer antibody testing and blood plasma donation. The patient’s only significant past medical history consisted of acquired hypothyroidism, which has remained controlled since the institution of levothyroxine in 2018. Her initial infection resulted in two weeks of acute symptoms, followed by spontaneous recovery. Week one consisted of classic “flu-like symptoms,” including fevers, chills, dry cough, headaches, mild dyspnea, and moderate fatigue with myalgias. At the start of week two, the first week’s symptoms subsided and were replaced with anosmia and ageusia, with complete resolution in four to five days thereafter.

The patient remained asymptomatic until mid-July 2020 when she abruptly began experiencing the symptoms, which eventually contributed to her long COVID diagnosis. At this initiation, the patient described a sudden and unprovoked burning sensation starting from her forehead and radiating through her entire scalp and down her neck. The sensation persisted for several hours before eventually dissipating and being followed by intense frontotemporal headaches, chest tightness with some dyspnea, palpitations and tachycardia with anxiety, dizziness with episodes of near-syncope on sitting or standing, and blurred vision. In the days following, the aforementioned symptoms would resume upon waking each morning and persist throughout the day. They were eventually coupled with significant fatigue, mild cognitive impairment or “brain fog” with impaired focus and memory recall, loss of appetite, diarrhea, uncharacteristic heat intolerance, and diffuse myalgias. The final symptom to appear during this cascade was severe, right-sided shoulder joint and/or muscle pain with radiations to the neck and right upper extremity, which led the patient to seek treatment.

Due to her history of hypothyroidism and concerns of exogenous thyrotoxicosis secondary to levothyroxine treatment, the patient initially sought care from endocrinology but was found to be euthyroid. She was later directed to primary care for the management of her right shoulder pain, at which she endorsed uncharacteristically extensive time spent outdoors in wooded areas of northern New Jersey prior to symptom onset due to the social distancing protocols of the time. A comprehensive workup was then completed with additional considerations for autoimmune or infectious etiologies. This workup included a complete blood count (CBC), comprehensive metabolic panel (CMP), lipid panel, hemoglobin A1c, erythrocyte sedimentation rate (ESR), C-reactive protein (CRP), rheumatoid factor, antinuclear antibodies (ANA) with reflex, vitamin B12, vitamin D, and a Lyme disease antibody panel. All results were unremarkable except for the Lyme disease antibody panel, which contained two positive bands (41 KD IgG and 23 KD IgM) and led the patient to be referred to infectious disease for further workup. Despite the lack of the minimum five positive bands on the antibody panel to support a Lyme disease diagnosis, the patient's clinical presentation led primary care and infectious disease to agree on initiating treatment. In early August 2020, she completed a two-week regimen of doxycycline 100 mg twice a day for treatment of a perceived early-disseminated Lyme disease infection. At this time, she was also able to more easily obtain outpatient COVID-19 testing and was found to still be both polymerase chain reaction (PCR)-positive and antibody-positive for the virus in July 2020 and September 2020. (Of note, COVID-19 titer quantification was not available to the general public at this time.)

Following the completion of the doxycycline regimen, the patient noted no improvement in her symptoms other than a mild reduction in her right shoulder pain. Furthermore, at treatment completion, she endorsed worsening blurred vision with the introduction of bilateral floaters, worsening of palpitations with tachycardia and/or anxiety, worsened heat intolerance, and an increased frequency of near-syncopal events, which all interfered with all physical activity. Her headaches remained near-constant and began alternating between tension-like and left-sided, migraine-like presentations with concurrent nausea and left-sided allodynia of the scalp. In the weeks thereafter, these symptoms were then coupled with new-onset sleep disturbances with insomnia and intermittent night terrors. In addition, the patient’s previously recovered olfactory sense was replaced with strong phantosmia. Per the patient, this caused the smells of otherwise benign foods, perfumes, or body odors to be swapped with foul smells of “burning rubber” or “rotting meat.” Lastly, the patient endorsed new-onset oligomenorrhea, with her menstrual cycles ranging from 60-70 days since her initial infection and her longest and most current cycle at the time lasting 82 days.

As her symptoms continually failed to improve, the patient sought care from a variety of specialists throughout the final months of 2020. Infectious disease ruled out several tick-borne illnesses through negative results of *Babesia*, *Anaplasma*, and repeat Lyme disease serology, as well as obtained negative *Giardia*, *Entamoeba histolytica*, and *Cryptosporidium* sampling. Neurology completed an extensive workup of her headaches including brain magnetic resonance imaging (MRI) without contrast but offered no findings. She was initially diagnosed with migraine-like headaches but found no benefit from common abortive therapies, including triptans and calcitonin gene-related peptide (CGRP) antagonists. She was eventually re-diagnosed with a novel “post-COVID headache” and found some relief with treatment like that for tension-type headaches, including Excedrin or Fioricet as needed for pain and prochlorperazine for nausea. Endocrinology performed a more extensive workup for fatigue and oligomenorrhea, including a repeat CBC, CMP, lipid panel, glycosylated hemoglobin (HbA1C), thyroid function panel, vitamin D level, and urinalysis, as well as morning cortisol, fasting insulin, follicle-stimulating hormone (FSH), luteinizing hormone (LH), estradiol, testosterone, dehydroepiandrosterone sulfate (DHEA-S), prolactin, iron panel with total iron binding capacity (TIBC) and ferritin, and vitamin B12 with folate. They also had the patient complete a 14-day continuous blood glucose monitoring protocol by placing a FreeStyle Libre 14-day device (Abbott Laboratories, Chicago, IL) intramuscularly. By January 2021, a diagnosis of polycystic ovarian syndrome (PCOS) with persistent nocturnal hypoglycemia was reached based on results from the continuous glucose monitoring, a positive LH:FSH ratio of 1.77, and her documented oligomenorrhea. Metformin escalation treatment was initiated alongside diet modifications, which led to a moderate reduction in cycle length and morning hypoglycemic symptoms. In February 2021, cross-conferencing between endocrinology, primary care, and neuromuscular medicine led to a formal diagnosis of post-acute COVID-19 syndrome or long COVID, and provided a formal referral to a comprehensive post-COVID care center. Upon evaluation at the post-COVID care center, no residual pulmonary deficits were noted on chest imaging and pulmonary function tests (PFTs), but a new-onset right bundle branch block with occasional premature ventricular contractions was noted on multiple ECGs. However, after a negative echocardiography (ECHO), exercise stress test, and sleep study, no intervention was indicated.

With supervision from her primary care provider, post-COVID care team, and medical school faculty, the patient simultaneously attempted her own self-rehabilitation. Through extensive trial and error and anecdotal evidence from programs designed for POTS rehabilitation, she constructed an exercise program using horizontally designed cardio equipment (i.e., reclined stationary bicycles, rowing machines, etc.) with extensive rest periods before, during, and after exercise. She also incorporated graded compression stockings and breathing exercises into both exercise and daily activities to lessen episodes of palpitations and/or tachycardia. In addition, she sought osteopathic manipulative treatment (OMT) through her faculty, which provide a moderate reduction in the severity and frequency of associated myalgias, headaches, and scalp allodynia. A variety of anxiety reduction techniques and sleep hygiene improvements were also employed with limited success in reducing her fatigue and brain fog.

By November 2020, the patient’s symptoms began to gradually improve, with notable reductions in palpitations, anxiety, and fogginess, but with a compensatory increase in fatigue and new-onset constipation and hair loss. The symptom pattern switched from a near-constant symptom presentation to a cyclic presentation with clear triggers associated with physical or mental overexertion; however, the threshold for “symptom relapse” increased over time. In January 2021, the patient completed the two-dose Moderna mRNA COVID-19 vaccination course and noted moderate improvements in her fatigue and brain fog in the first 24 hours following her first dose. Her headaches and scalp allodynia eventually receded as well nearly six months later in August 2021. To date, the patient identifies as having made a full recovery from long COVID and only endorses residual blurred vision managed with a stronger vision prescription and reduced but continued oligomenorrhea and hypoglycemic sensitivity managed with diet, exercise, and metformin treatment. She has since been reinfected twice with SARS-CoV-2 in July 2022 and January 2023 and received molnupiravir for the first of these two reinfections but spontaneously recovered both times without relapse of any of her long COVID symptoms.

## Discussion

The case presented details the uniquely subjective and varied disease process of long COVID, thereby continuing the need for physicians to evaluate it as a diagnosis of exclusion. However, the anecdotal evidence detailed here of this eventual diagnosis suggests a phasic approach to its progression. The “pre-phase” or “Phase 0” consists of the initial SARS-CoV-2 infection, with either an asymptomatic presentation or classic COVID-19 symptoms, i.e., flu-like symptoms with or without loss of taste and smell. “Phase 1” represents the acute hyperinflammatory response associated with the development of “hyperactive” long COVID symptoms. In the case presented and other anecdotal accounts, the symptoms typically developed within weeks to months following the initial infection; however, they can also present as a continuation of specific symptoms from the initial infection. Cardinal symptoms may include headaches, palpitations with or without tachycardia, unprovoked anxiety, dizziness and/or near-syncope, blurred vision, brain fog with or without memory deficits, diarrhea and/or increased gastric motility, losses of appetite, heat intolerance, acute myalgias, and new-onset phantosmia.

**Figure 1:**
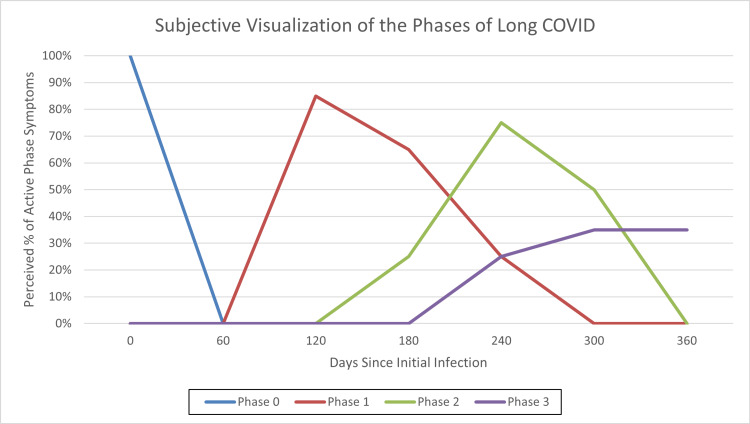
A subjective visualization of the perceived phases of long COVID symptomology, based on the case presented.

“Phase 2” represents a perceived reduction in hyperinflammation with the emergence of “hypoactive” long COVID symptoms. As presented in this case, these symptoms appear several months following the first phase, with clear shifts from hyperactive to hypoactive symptoms in several body systems. Such shifts can include a compensatory increase in fatigue and a decrease in anxiety and/or palpitations, a decrease in gastric motility and/or constipation, telogen effluvium, and more diffuse or generalized myalgias. The final phase, “Phase 3,” consists of a gradual recession of some “hypoactive” symptoms and the emergence of seemingly permanent deficits. The switch between the second and third phases is more obscure in practice and can occur over several months to years with intermittent periods of symptom relapse. At this point, the prognosis of one’s auto-recovery versus a limited recovery may be clearer to both patients and providers and can guide futility assessments of symptomatic treatments or interventions. A subjective visualization of the interplay of these phases is provided in Figure 1 below.

Although long COVID shares myriad symptoms with other well-known post-viral illnesses, the illness itself exists as a distinct diagnosis with unique symptoms, including phantosmia and a potentially unpredictable onset depending on one’s transition from “Phase 0” to “Phase 1.” In addition, SARS-CoV-2 post-viral shedding commonly occurs for greater periods of time in the long COVID population [5]. This persistent PCR-positivity may correlate with the onset of long COVID symptoms and thereby have the potential as a predictive or diagnostic value in this patient population. Nevertheless, long COVID will remain a clinical diagnosis following an exclusionary workup for the foreseeable future.

The greatest improvements in a patient’s long COVID prognosis seemingly come from allowing for the passage of time and support of an extended healing period. Concepts such as “radical rest” or “pacing” have entered the long COVID conversation as both a preventative and restorative concept to allow for ample healing and prevent symptom exacerbations, regardless of the presence of post-exertional malaise [6]. These techniques should be considered in conjunction with mRNA-based COVID-19 vaccination administration, which is believed to be both preventative and therapeutic in the long COVID setting [7]. Thereafter lies utility in treating secondary illness that may have been exacerbated by SARS-CoV-2 infection or associated deconditioning and encouraging a variety of lifestyle changes to adapt to this otherwise chronic illness.

The benefit of this account lies not only in its detailed recollection of symptomology but in its availability of trial-and-error data in self-studied treatment options for clinician reference. Although the aforementioned reliance on the passage of time and vaccination proved most beneficial, several symptomatic treatments were also endorsed by the patient. These include graded horizontal exercise therapy like that employed in the POTS patient population [8], as well as breathing techniques and graded compression stocking usage during exercise, daily activities, and periods of symptom exacerbation. (Of note, in this case, the patient’s compression stocking usage only provided temporary benefit and over time became “overly compressive” and seemingly contributed to several symptom re-exacerbations.) Multiple sessions of OMT were effective in reducing the patient’s myalgias and any headaches present at the time of treatment and provided useful meditative and stretching strategies for at-home pain reduction. Abortive therapies for tension-type headaches with nausea (i.e., Excedrin, Fioricet, and prochlorperazine) in combination with increases in caffeine and salt consumption with increased fluid intake helped managed associated headache pain, orthostatic-related palpitations, and some aspects of fatigue and brain fog. The patient was also able to access several digital interventions with moderate symptom alleviation, including self-guided smell re-training for phantosmia and a clinical trial evaluating therapeutic gaming for mild cognitive impairment (NCT04843930). Lastly, an increased prioritization of her mental health through cognitive behavioral therapy and a strong network of peers within and outside of medicine, as well as sleep hygiene through improvements in her pre-sleep environment and reduction of nocturnal hypoglycemic episodes both made small but recognizable differences in her fatigue and cognition.

Several ineffective interventions were noted by the patient, with the most prominent being traditional exercise rehabilitation (i.e., vertical cardio training via treadmill or elliptical, or strength training with excessive straining), as this would reliably exacerbate her fatigue to varying degrees. In addition, not only were traditional abortive therapies for migraine ineffective in managing her symptoms, but the introduction of a CGRP antagonist resulted in a moderate increase in fatigue and brain fog for the duration of treatment. Although likely unrelated, it is also worth noting that her doxycycline regimen for presumed early disseminated Lyme disease contributed to a subjective worsening of blurred vision, heat intolerance, and near-syncopal events. Finally, the patient attempted additional self-treatment following anecdotal recommendations of a variety of supplements, including vitamin C, vitamin D, vitamin B12, a generalized B-complex vitamin, n-acetyl cysteine (NAC), and turmeric extract. None of these supplements provided her with any discernable benefit and NAC led to a subjective increase in headache frequency and duration.

A strong patient-provider relationship is essential to the success of comprehensive long COVID treatment. Many patients are still routinely dismissed by the medical community over concerns of malingering or lack of knowledge about management options. The patient presented here is health literate and has access to seemingly high-quality healthcare, yet endorsed several instances where her symptoms were ignored, misdiagnosed, or misattributed to perceived anxiety by several clinicians. Because long COVID exists as a clinical, exclusionary diagnosis with limited ability to differentiate the effects of the syndrome versus deconditioning, it is critical for all healthcare providers to recognize and adequately treat it to avoid further deconditioning and reductions in quality of life [9]. Furthermore, considering the plausible increase in depression and suicide risk in the long COVID population [10], these patients deserve attentive and evidence-based treatment regardless of the multifactorial causes of their illness. Engagement with cognitive behavioral therapy and online support communities has already shown some benefit in the reduction of psychosomatic symptom burden for these patients [11,12]. Additional educational initiatives for patients and providers, as well as advocacy for accommodations for those with more significant impairments, are necessary for the systemic support of this population.

Although the subjective nature of this case report limits its impact, it may still serve as a launch pad for both improved diagnostic understanding of long COVID and research into more targeted treatment options. Such options may include examining correlations between long COVID and all metabolic syndromes, including PCOS, and the subsequent therapeutic benefit of improved insulin control through metformin treatment [13]. Exploring the greater interplay of long COVID and autoimmunity both in general and with respect to estrogen modulation may support causational theoretical models linking the illness with POTS, ME/CFS, MCAS, and other post-viral illness syndromes [14]. Through such models, targeted anti-inflammatory or immunomodulatory treatments can also be trialed to ideally prevent or reduce long COVID sequelae in a more controlled manner. The influence of co-infections, such as the Lyme disease infection described here, or other accounts of Epstein-Barr virus or other herpesviruses infection or reactivation [4], should also be granted further consideration in the syndrome’s pathogenesis and management. Finally, the triumphs in anti-viral therapy for acute COVID-19 infection may have not only potentially protected this patient from symptom relapse following re-infection but are highly favorable avenues for long COVID treatment investigation [15].

## Conclusions

As the COVID-19 pandemic is reduced to endemicity, those suffering from long COVID will continue their emergence as a distinct patient population in need of improved diagnosis and management. In the case of this 26-year-old female, the unfortunate early timing of her illness left her unable to seek comprehensive long COVID care, as it did not yet exist. However, as scientific bodies mount ample evidence of the existence and impact of the long COVID syndrome, it is imperative for clinicians to adapt and improve the delivery of care accordingly. Such initiatives should include support for the creation of additional post-COVID care centers with an understanding of “pacing” and POTS or dysautonomia rehabilitation. Logistically, improving the recognition of long COVID beyond these post-COVID centers would conceivably reduce excess expenditure on unnecessary examinations while guiding patients earlier in their disease to more beneficial resources, such as physical therapy, occupational therapy, and/or OMT. Ultimately, the legitimization of long COVID as a clear and present potential consequence of an acute COVID-19 infection through the continual collection of anecdotal and statistical data will inevitably improve the delivery of care by bestowing it with the significance it deserves as a chronic but manageable illness.
